# Supplementary material for: An improved formulation of human neural stem cells for the treatment of geographic atrophy
Source: PLoS One. 2026 Aug 10;21(8):e0355552. doi: 10.1371/journal.pone.0355552 (PMC13456245; doi:10.1371/journal.pone.0355552)
Supplement: Supplementary file 10002 [file pone.0355552.s002.pdf]

PLOS One

An improved formulation of human neural stem cells for the treatment of geographic atrophy

--Manuscript Draft--

**Manuscript Number:**	PONE-D-25-67955
**Article Type:**	Research Article
**Full Title:**	An improved formulation of human neural stem cells for the treatment of geographic atrophy
**Short Title:**	An improved formulation of human neural stem cells for the treatment of geographic atrophy
**Corresponding Author:**	Renee C. Ryals, Ph.D.<br>Oregon Health & Science University Casey Eye Institute<br>Portland, UNITED STATES OF AMERICA
**Keywords:**	human neural stems cells; geographic atrophy; RCS rat; neuroprotection
**Abstract:**	Purpose: This study aimed to compare the neuroprotective effects of subretinally delivered human central nervous system stem cells (HuCNS-SC) and a novel, single-cell suspension formulation, Neubright (NB) in the RCS rat. Methods: At postnatal day 21, RCS rats were subretinally injected with a 150,000 cell dose of either HuCNS-SC or NB in a 2 µL volume. The contralateral control eye was injected with balanced salt solution. Visual function outcomes were measured using optokinetic tracking and electroretinography. Whole globes were harvested at P60 and P180 for immunofluorescence (IF). To identify cells in the subretinal space, retinal cross-sections were stained with a human specific marker (STEM121). Cell spread, outer nuclear layer (ONL) thickness, and cell migration were quantified using confocal microscopy and image J. Results: IF demonstrated a clear presence of STEM121-positive cells in the subretinal space after delivery of both HuCNS-SC and NB. ONL thickness was increased in areas adjacent to the injected cells, whereas progressive degeneration was observed outside of treated areas. The novel NB formulation had significantly increased cell spread and visual performance compared to the original HuCNS-SC formulation.Conclusions: A novel single-cell suspension of human neural stem cells, NB, with high cell viability was successfully generated. NB improved cell spread in the subretinal space compared to HuCNS-SC improving visual outcomes in the RCS rat.Translational Relevance: This bridging study will be used as an investigational new drug approval enabling study to evaluate NB in clinical trials for macular retinal degeneration.
**Order of Authors:**	Sebastian E. Arrizabalaga
	Nobuko Uchida
	Allison Curtis
	Ann Tsukamoto
	Theodore Leng, MD
	Renee C. Ryals, Ph.D.
**Additional Information:**	
**Question**	**Response**
**Financial Disclosure**<br><br>Enter a financial disclosure statement that describes the sources of funding for the work included in this submission. Review the submission guidelines for detailed requirements. View published research	Yes

Powered by Editorial Manager® and ProduXion Manager® from Aries Systems Corporation

articles from *PLOS ONE* for specific examples.<br><br>This statement is required for submission and **will appear in the published article** if the submission is accepted. Please make sure it is accurate.<br><br>**Funded studies**<br>Enter a statement with the following details:<br>• Initials of the authors who received each award<br>• Grant numbers awarded to each author<br>• The full name of each funder<br>• URL of each funder website<br>• Did the sponsors or funders play any role in the study design, data collection and analysis, decision to publish, or preparation of the manuscript?<br><br>Did you receive funding for this work?	
Please add funding details.<br>as follow-up to "**Financial Disclosure**<br><br>Enter a financial disclosure statement that describes the sources of funding for the work included in this submission. Review the submission guidelines for detailed requirements. View published research articles from *PLOS ONE* for specific examples.<br><br>This statement is required for submission and **will appear in the published article** if the submission is accepted. Please make sure it is accurate.	This work was supported by CIRM TRAN1-11300 (TL, RCR), NIH grant P30-EY026877 (TL), NIH P30 EY010572 core grant, the Malcolm M. Marquis, MD Endowed Fund for Innovation, and an unrestricted grant from Research to Prevent Blindness (TL, RCR). BOCO Silicon Valley provided the hNSCs.

**Funded studies**<br>Enter a statement with the following details:<br>• Initials of the authors who received each award<br>• Grant numbers awarded to each author<br>• The full name of each funder<br>• URL of each funder website<br>• Did the sponsors or funders play any role in the study design, data collection and analysis, decision to publish, or preparation of the manuscript?<br><br>Did you receive funding for this work?"	
Please select the country of your main research funder (please select carefully as in some cases this is used in fee calculation).<br>as follow-up to "**Financial Disclosure**<br><br>Enter a financial disclosure statement that describes the sources of funding for the work included in this submission. Review the submission guidelines for detailed requirements. View published research articles from *PLOS ONE* for specific examples.<br><br>This statement is required for submission and **will appear in the published article** if the submission is accepted. Please make sure it is accurate.<br><br>**Funded studies**<br>Enter a statement with the following details:<br>• Initials of the authors who received each award<br>• Grant numbers awarded to each author<br>• The full name of each funder<br>• URL of each funder website<br>• Did the sponsors or funders play any role in the study design, data collection and analysis, decision to publish, or preparation of the manuscript?<br><br>Did you receive funding for this work?"	UNITED STATES - US
**Competing Interests**	The authors have declared that no competing interests exist.

Use the instructions below to enter a competing interest statement for this submission. On behalf of all authors, disclose any competing interests that could be perceived to bias this work—acknowledging all financial support and any other relevant financial or non-financial competing interests.<br><br>This statement is required for submission and **will appear in the published article** if the submission is accepted. Please make sure it is accurate and that any funding sources listed in your Funding Information later in the submission form are also declared in your Financial Disclosure statement.<br><br>View published research articles from *PLOS ONE* for specific examples.<br><br>**NO authors have competing interests**<br><br>Enter: *The authors have declared that no competing interests exist.*<br><br>**Authors with competing interests**<br><br>Enter competing interest details beginning with this statement:<br><br>*I have read the journal's policy and the authors of this manuscript have the following competing interests: [insert competing interests here]*<br><br>* typeset	
**Ethics Statement**<br><br>Enter an ethics statement for this submission. This statement is required if the study involved:<br><br>• Human participants<br>• Human specimens or tissue<br>• Vertebrate animals or cephalopods<br>• Vertebrate embryos or tissues	Approval for all experiments was granted by the Institutional Animal Care and Use Committee at OHSU (IACUC Protocol number TR02_IP00001387) and adhered to the ARVO Statement for the Use of Animals in Ophthalmic and Vision Research.

• Field research<br><br>Write "N/A" if the submission does not require an ethics statement.<br><br>General guidance is provided below. Consult the submission guidelines for detailed instructions. **Make sure that all information entered here is included in the Methods section of the manuscript.**	

Powered by Editorial Manager® and ProduXion Manager® from Aries Systems Corporation

**Format for specific study types**<br><br>**Human Subject Research (involving human participants and/or tissue)**<br>• Give the name of the institutional review board or ethics committee that approved the study<br>• Include the approval number and/or a statement indicating approval of this research<br>• Indicate the form of consent obtained (written/oral) or the reason that consent was not obtained (e.g. the data were analyzed anonymously)<br><br>**Animal Research (involving vertebrate animals, embryos or tissues)**<br>• Provide the name of the Institutional Animal Care and Use Committee (IACUC) or other relevant ethics board that reviewed the study protocol, and indicate whether they approved this research or granted a formal waiver of ethical approval<br>• Include an approval number if one was obtained<br>• If the study involved *non-human primates*, add *additional details* about animal welfare and steps taken to ameliorate suffering<br>• If anesthesia, euthanasia, or any kind of animal sacrifice is part of the study, include briefly which substances and/or methods were applied<br><br>**Field Research**<br><br>Include the following details if this study involves the collection of plant, animal, or other materials from a natural setting:<br>• Field permit number<br>• Name of the institution or relevant body that granted permission	
**Data Availability**<br><br>Authors are required to make all data underlying the findings described fully available, without restriction, and from the time of publication. PLOS allows rare exceptions to address legal and ethical concerns. See the PLOS Data Policy and FAQ for detailed information.	Yes - all data are fully available without restriction

A Data Availability Statement describing where the data can be found is required at submission. Your answers to this question constitute the Data Availability Statement and **will be published in the article**, if accepted.<br><br>**Important:** Stating 'data available on request from the author' is not sufficient. If your data are only available upon request, select 'No' for the first question and explain your exceptional situation in the text box.<br><br>Do the authors confirm that all data underlying the findings described in their manuscript are fully available without restriction?	
**Describe where the data may be found in full sentences. If you are copying our sample text, replace any instances of XXX with the appropriate details.**<br><br>• If the data are **held or will be held in a public repository**, include URLs, accession numbers or DOIs. If this information will only be available after acceptance, indicate this by ticking the box below. For example: *All XXX files are available from the XXX database (accession number(s) XXX, XXX.).*<br>• If the data are all contained **within the manuscript and/or Supporting Information files**, enter the following: *All relevant data are within the manuscript and its Supporting Information files.*<br>• If neither of these applies but you are able to provide **details of access elsewhere**, with or without limitations, please do so. For example:<br><br>*Data cannot be shared publicly because of [XXX]. Data are available from the XXX Institutional Data Access / Ethics Committee (contact via XXX) for researchers who meet the criteria for access to confidential data.*<br><br>*The data underlying the results presented in the study are available from (include the name of the third party*	All relevant data are within the manuscript and its Supporting Information files.

and contact information or URL).<br>• This text is appropriate if the data are owned by a third party and authors do not have permission to share the data.<br><br>* typeset	
Additional data availability information:	

Powered by Editorial Manager® and ProduXion Manager® from Aries Systems Corporation

Manuscript

Click here to access/download;Manuscript;Manuscript File PLOS one Final.docx

An improved formulation of human neural stem cells for the treatment of geographic atrophy

Sebastian E. Arrizabalaga[1], Nobuko Uchida[2], Allison Curtis[1], Ann Tsukamoto[2], Theodore Leng[2], Renee C. Ryals[1*]

[1]Ophthalmology, Oregon Health & Science University Casey Eye Institute, Portland, OR, United States
[2]Byers Eye Institute at Stanford, Stanford University School of Medicine, Palo Alto, CA, United States

This work was supported by CIRM TRAN1-11300 (TL, RCR), NIH grant P30-EY026877 (TL), NIH P30 EY010572 core grant, the Malcolm M. Marquis, MD Endowed Fund for Innovation, and an unrestricted grant from Research to Prevent Blindness (TL, RCR). BOCO Silicon Valley provided the hNSCs.

There are no commercial relationships to disclose.

*Corresponding author

Email: ryals@ohsu.edu

ABSTRACT

Purpose: This study aimed to compare the neuroprotective effects of subretinally delivered human central nervous system stem cells (HuCNS-SC) and a novel, single-cell suspension formulation, Neubright (NB) in the RCS rat.

Methods: At postnatal day 21, RCS rats were subretinally injected with a 150,000 cell dose of either HuCNS-SC or NB in a 2 μL volume. The contralateral control eye was injected with balanced salt solution. Visual function outcomes were measured using optokinetic tracking and electroretinography. Whole globes were harvested at P60 and P180 for immunofluorescence (IF). To identify cells in the subretinal space, retinal cross-sections were stained with a human specific marker (STEM121). Cell spread, outer nuclear layer (ONL) thickness, and cell migration were quantified using confocal microscopy and image J.

Results: IF demonstrated a clear presence of STEM121-positive cells in the subretinal space after delivery of both HuCNS-SC and NB. ONL thickness was increased in areas adjacent to the injected cells, whereas progressive degeneration was observed outside of treated areas. The novel NB formulation had significantly increased cell spread and visual performance compared to the original HuCNS-SC formulation.

Conclusions: A novel single-cell suspension of human neural stem cells, NB, with high cell viability was successfully generated. NB improved cell spread in the subretinal space compared to HuCNS-SC improving visual outcomes in the RCS rat.

Translational Relevance: This bridging study will be used as an investigational new drug approval enabling study to evaluate NB in clinical trials for macular retinal degeneration.

INTRODUCTION

Age-related macular degeneration (AMD) is a progressive, degenerative disease and the most common cause of blindness in developed countries.[1] Two primary forms of advanced AMD have been identified: neovascular (exudative) and non-neovascular (nonexudative). About 90% of AMD patients have the nonexudative form characterized by drusen formation within the macula.[2] Late stage nonexudative AMD, referred to as geographic atrophy (GA-AMD), features degeneration of the retinal pigment epithelial (RPE) cells followed by the loss of the light-sensitive photoreceptor cells, leading to central vision loss.[2] Currently, there are two FDA approved therapies for GA-AMD. However these complement inhibitors have limited efficacy and undesired side effects; thus alternative therapeutic approaches are needed.[3,4]

Therapeutic development for GA-AMD has included immunomodulation, visual cycle modulation, neuroprotection, and cell-based therapies.[5] Since their isolation in 2000, human central nervous system stem cells (HuCNS-SC) grown as neurospheres have been in clinical development as a neuroprotective agent to slow the progression of GA-AMD.[6] HuCNS-SC are self-renewing, multilineage-producing cells that hold great promise for treating a large number of neurodegenerative disorders.[7–10] They were established by isolating CD133+ cells from human fetal brain tissue, which are highly enriched for neurosphere-initiating cells with self-renewal capabilities at the single-cell level. Based on these initial characterizations, individual HuCNS-SC banks have been successfully established by directly isolating HuCNS-SC with high levels of CD133, but little or no CD24 (CD133+/CD24-/lo) from single donated human fetal brain tissues (16–20 weeks gestation). These cells undergo selective propagation in a defined serum-free

culture media, are grown as neurospheres, and can be cryopreserved in cell banks[6]. Previous studies have shown that HuCNS-SC are capable of proliferation, migration, and multilineage differentiation in a site-appropriate manner when transplanted into brains or spinal cord of immunodeficient mice, thereby recapitulating temporal development of HuCNS-SC from the human fetal brain. These banked cells continue to express HuCNS-SC markers such as CD133 and Sox2. Additionally, they have biological HuCNS-SC activities with multiple mechanisms of actions, providing neuroprotection, myelination, and retinal preservation via site-appropriate global migration.[7–10]

Banked HuCNS-SC have been transplanted into the Royal College of Surgeons (RCS) rat model, a well-established model used to evaluate cellular therapeutics for AMD.[11–13] Previous studies have shown that HuCNS-SC survived for at least 8 months after transplantation into the subretinal space of P21 RCS rats, where they preserved host photoreceptors from degeneration, and minimized the loss of vision long-term.[11] The mechanism of action for this benefit was multifactorial, but included the ability of HuCNS-SC cells to phagocytose photoreceptor outer segments and to significantly increase host RPE cell proliferation.[12,13]

These successful pre-clinical studies led to an FDA-approved Phase I/II Study of the Safety and Preliminary Efficacy of Human Central Nervous System Stem Cells (HuCNS-SC) Subretinal Transplantation in Subjects With Geographic Atrophy of Age-Related Macular Degeneration (NCT01632527).[14] This was an open-label dose-escalation investigation of unilateral subretinal transplantation of HuCNS-SC cells (250,000 to 1 million) in subjects with GA-AMD. Cells were delivered superotemporal to

the fovea near the junctional zone, outside the area of GA-AMD.[14] Results from these studies showed that HuCNS-SC transplantation showed a significantly slower progression rate compared to untreated fellow eyes (0.29 ± 0.58 mm vs. 1.08 ± 0.65 mm; P = 0.007), but only in the superotemporal area of the GA-AMD lesion.[14] Since the delivery of the cells was superotemperal of the GA-AMD lesion and that was the only area of the GA-AMD lesion positively impacted by the cells, we hypothesize that treating a larger area adjacent to the GA-AMD lesion would improve outcomes.

To achieve this, first, multiple subretinal detachments around the entire GA-AMD lesion may be required. Additionally, we hypothesized that generating a single-cell formulation would increase cell spread in the subretinal space increasing the treated area. Here, we describe a novel, single-cell suspension of HuCNS-SC called Neubright (NB) and evaluated its ability to slow retinal degeneration in the RCS rat. We showed that NB increased cell spread in the subretinal space and performed on par or better than HuCNS-SC in visual outcomes. This bridging study comparing the neuroprotective effects achieved with HuCNS-SC and NB will be used in as an IND enabling study to evaluate NB in clinical trials for macular retinal degeneration.

METHODS

Animals

Breeder Royal College of Surgeons (RCS) rats were purchased from the Rat Resource & Research center (RRRC #315). Breeding and colony maintenance occurred at the Oregon Health & Science University, Casey Eye Institute (OHSU, CEI). Approval for all experiments was granted by the Institutional Animal Care and Use Committee at OHSU

(IACUC Protocol number TR02_IP00001387) and adhered to the ARVO Statement for the Use of Animals in Ophthalmic and Vision Research.

Cell Preparation

Two different formulations were prepared for this transplantation study: HuCNS-SC, small clusters and NB, single-cells. Vials from a working cell bank (WCB) were thawed and cultured for 10 days in a DASbox bioreactor system (Eppendorf, Germany) in DMEM/F12 basal medium (ThermoFisher) with cytokines of recombinant human (rhu) fibroblast factor 2 (20 ng/mL), rhu epidermal growth factor (20 ng/mL), and rhu leukemia inhibitory factor (10 ng/mL). To generate small cell clusters of HuCNS-SC suitable for subretinal injection in RCS rats, neutrospheres were harvested on day 9, dissociated into single cells using TrypLE (ThermoFisher), and reseeded into the DASbox for one additional day to form the small cluster formulation by day 10. To generate a single-cell formulation of NB, neurospheres were harvested at day 10 and enzymatically dissociated into single-cells. The HuCNS-SC and NB formulations used in this study were derived from the Master Cell Bank (MCB 5016.M3), which was previously prepared for the GA-AMD clinical trial (clinical trial number NCT01632527).

Injections

RCS rats were weaned at postnatal day 20 (P20) and provided a supply of cyclosporine treated water. Animals were anesthetized using a ketamine (81 mg/kg) and xylazine (5 mg/kg) mixture and eyes were dilated with 2.5% phenylephrine and 0.5% tropicamide. Animals were then injected in one eye with either HuCNS-SC or NB at a dose of 150,000 cells in a 2 μL trans-scleral subretinal injection using a hand pulled glass

cannula attached to a Hamilton syringe.[15] The dose was validated by manual counting via a hemacytometer during cell preparation. Contralateral eyes were injected with balanced salt solution (BSS). Immune suppression post operation consisted of intraperitoneal injections of dexamethasone (1 mg/mL) every 48 hours for two weeks post op and ad libitum cyclosporine until harvest.

Cyclosporine Administration

At first rats were given a standard regimen of ad libidum cyclosporine A impregnated water at a dose of 210 mg/L. However, animals began to suffer health problems. Thus, animals were switched to an escalating dose scheme beginning with a dose of 52.5 mg/L from P21-P35, followed by a dose of 105 mg/L from P35-P150, and ending with a dose of 157.5mg/L from P150-P240. With this regimen, there were no adverse effects and animals were healthy to P240. To ensure blood cyclosporine concentration levels remained at the target therapeutic dose, 300 ng/mL, blood was collected at P35, P90, and P200 and sent to Auburn University for quantification.

Optokinetic Tracking (OKT)

OKT was evaluated at P60, P90, and P180 on a Cerebral Mechanics OptoMotry system to measure preservation of visual performance.[16] After system calibration, animals were placed in a low rimmed bowl attached to the testing pedestal and allowed to acclimate for one to two minutes to the testing environment before data collection. We used a simple staircase method at 100% contrast. No animal was tested for longer than 30 minutes on any given testing day, necessary retesting was performed on the following

day to reduce animal stress. Data for each eye at each timepoint was pooled by treatment group.

Electroretinography (ERG)

ERGs were performed on a custom-built ERG machine as previously described. RCS rats were dark adapted over-night prior to testing.[17] Dark adapted animals were sedated using a ketamine (81 mg/kg) and xylazine (5 mg/kg) mixture and eyes were dilated with 2.5% phenylephrine and 0.5% tropicamide. Animals were exposed to a single scotopic flash having a luminance of 3.55 log cd * s/m^2. A-wave and B-wave amplitudes were extracted from waveforms and averaged for each treatment group.

Immunofluorescence (IF)

Whole globes were harvested and dissected into eyecups at P60 and P180. Eyecups were fixed in 4% paraformaldehyde for 24 hours before being put through a sucrose gradient (10%, 20%, and 30% sucrose by weight for 24 hours each) to prepare for freezing in OCT compound. Frozen eyes were sectioned on a cryostat at a thickness of 12 microns. Tissue sections were stained with a marker specific to human cells (STEM121, cat#Y40410) and a nuclear counterstain (DAPI). For ONL thickness analysis, confocal images of retinal cross-sections from each group were taken at 10x magnification. Images were taken in areas within the treated retina and areas outside of the treated area. Using Image J, ONL thickness was quantified in the 10x images. Measurements for each eye and retinal location were averaged and pooled by treatment group before analysis. Additional 10x confocal images were taken to quantify cell spread. Sections containing the maximum treated area were selected and stained

for cell spread measurements. The width of STEM121+ cells were measured in the Leica confocal software suite. When the STEM121 positivity went beyond one 10x image several images were captured and composited to a single image and measurement.

Safety

A safety evaluation was performed assessing cell migration and proliferation. At P240, both whole eye and optic nerves were harvest from 10 animas resulting in 5 NB-treated samples, 5 HuCNS-SC-treated samples, and 8 BSS-injected control samples. Whole eyes were dissected, frozen and sectioned as previously described. Optic nerves were also frozen and sectioned for staining. Retinal cross-sections that contained the optic nerve head (ONH) and optic nerves were stained with a human cell marker (STEM121, cat#Y40410), cell proliferation marker (Ki-67, cat#AB16667), and nuclear counterstain (DAPI). Stained sections were imaged by confocal microscopy and the number of cells were counted.

Statistics

All statistical analysis was performed in GraphPad Prism 10. P-values of less than 0.05 were considered significant for all tests. For OKT, spatial frequencies were obtained for each eye and grouped by treatment group. Treatment groups were compared using a 2-way ANOVA mixed-effects analysis with Tukey's multiple comparisons test. Cell spread achieved with HuCNS-SC and NB was compared using an unpaired t-test. ONL thickness measurements and a-wave and b-wave amplitudes were grouped by treatment and analyzed with a one-way ANOVA with Tukey's multiple comparisons test.

RESULTS

Cell characterization

Two formulations, HuCNS-SC (clusters) and NB (single-cell), were characterized for purity (CD133) and potency/identity (Sox2). CD133 is a surface marker expressed on neural stem cells and was used to isolate HuCNS-SC from human brain tissue. Upon expansion, $CD133^+$ cells retain the self-renewal and multipotent characteristics of banked HuCNS-SC. Sox2 is a transcription factor expressed in neural stem cells and is downregulated as the cells differentiate. Therefore, Sox2 expression serves as an indicator of self-renewal capacity and neural stem cell potency. Flow cytometry (FACS) analysis showed that both formulations exhibited high expression levels, with average of over 95% of cells positive for CD133 and over 90% positive for Sox2 (**Figure S1C**). A cluster-cell morphology was observed for the HuCNS-SC formulation, whereas the NB formulation showed a distinct single-cell morphology when imaged on a hemocytometer (**Figure S1A&B**).

Early Assessments

RCS rats underwent successful trans-scleral subretinal surgeries delivering 150,000 HuCNS-SC or NB cells to the subretinal space. Untreated RCS rats undergo rapid retinal degeneration.[18] By 3 weeks of age, photoreceptor outer segments show evidence of disruption with the development of an apical debris zone. At 7 weeks, approximately 50% of the photoreceptor nuclei degenerate. By 12 weeks, the outer nuclear layer reduces to a single layer of photoreceptor cell bodies and the debris zone occupies the former outer segment area.[18] To assess successful transplantation, some

eyes were harvested at an early P60 (~8 weeks of age) time point, approximately 5-6 weeks after injection. Cells, stained with STEM121, were successfully located in the superotemporal or superonasal subretinal space in both HuCNS-SC and NB-treated eyes (**Figure 1A**). Cells were not observed in untreated areas of the retina or in BSS-injected control eyes. To determine neuroprotection achieved, ONL thickness was quantified in acquired retinal-cross sections. At P60, both formulations showed significant rescue above the BSS-injected controls as well as untreated areas in treated eyes (**Figure 1B**). NB treated ONL thickness averaged 12 cell bodies and HuCNS-SC averaged 10 cell bodies compared to outside the treated areas and controls which averaged between 5-7 cell bodies thick (**Figure 1B,** $p<0.05$).

When observing the cell morphology in the subretinal space, we found that the cluster formulation, HuCNS-SC, stayed in large spherical clumps, whereas the single-cell suspension, NB, formed a thinner more elongated group of cells (**Figure 1A**). We then sought to quantify the cell spread of the two different formulations at P60. NB on average covered a 1,097 μm width of the neural retina, whereas HuCNS-SC covered an average a 523 μm width of the neural retina. Using a single-cell suspension formulation, NB, cell spread was increased by 2-fold (**Figure 1C,** $p<0.05$).

Fig 1. NB preserved ONL thickness and increased cell spread 5 weeks post-injection.

(A) Confocal images of the retina at P60 show presence of transplanted cells in the subretinal space (red, STEM121) as well as the preservation of ONL in treated areas of the retina when compared to retina outside the treated area or retina from BSS-injected

eyes. (B) ONL thickness measurements showing efficacy of both treatments at P60. Measurements of ONL thickness indicate an increased number of photoreceptors in areas of the retina adjacent to transplanted cells compared to untreated areas indicative of protective effects of the transplanted cells. Statistical analysis was performed with a one-way ANOVA with Tukey's multiple comparisons test. Sample sizes: Control n=5, HuCNS-SC untreated n=4, HuCNS-SC treated n=4, NB untreated n=15, NB treated n=15. (C) Representative confocal images demonstrate cell spread in the subretinal space of RCS rats at P60. When cell spread was quantified, the NB formulation was able to increase cell spread (the treated area) by 2-fold. Measurements were taken from 3 HuCNS-SC® treated eyes and 11 NB treated eyes. Analysis was performed with a paired t-test.

Long-term Assessments

With the positive IF data, we continued our studies out to P180. Animals were assessed for visual performance via optokinetic tracking (OKT) at P30, P60, P90 and P180 (**Figure 2**). Spatial frequencies were increased in both treatment groups through P90 with NB eyes averaging 0.432 cycles/degree (c/d) and HuCNS-SC eyes averaging 0.390 c/d compared to 0.365 c/d for BSS-injected control eyes. By P180, the efficacy of the cluster formulation, HuCNS-SC, declined not showing a significant difference compared to the control (HuCNS-SC - 0.223 c/d, BSS - 0.179 c/d, ns). In contrast, the NB group with a 0.350 c/d had a significantly higher visual performance than both the BSS-injected group and HuCNS-SC group at P180 (**Figure 2,** $p<0.05$).

Fig 2. NB maintained visual performance for 22 weeks post-injection.

Longitudinal OKT data showing better visual performance of NB treated eyes compared to HuCNS-SC® and BSS-injected control eyes. At P180, NB-injected eyes had significant improvements in visual performance compared to all other groups. Sample sizes for each timepoint are listed above the corresponding time. Statistical analysis was performed with a 2-way ANOVA mixed-effects analysis with Tukey's multiple comparisons test.

To investigate if visual performance gains associated with presence of cells in the subretinal space, some of these eyes were harvested at P180 and ONL thickness was quantified in acquired retinal-cross sections (**Figure 3**). At P180, cells were present in the subretinal space and both cell-treated groups maintained significant increases in ONL thickness compared to the untreated areas. ONL thickness in NB-treated areas averaged 2 cell bodies, which was on par with HuCNS-SC treated areas, about 3 cell bodies (**Figure 3B, p<0.05**). Areas outside the treated area as well as the BSS injected control averaged 0 cell bodies thick, having lost all photoreceptors.

Fig 3. NB preserved ONL thickness on par with HuCNS-SC at 22 weeks post-injection.

(A) Confocal images of the retina at P180 show presence of transplanted cells in the subretinal space (red, STEM121) as well as the preservation of ONL in treated areas of the retina when compared to retina outside the treated area. (B) ONL thickness measurements showing efficacy of both treatments at P180. Measurements of ONL thickness indicate an increased number of photoreceptors in areas of the retina adjacent to transplanted cells compared to untreated areas indicative of protective effects of the transplanted cells. Statistical analysis was performed with a one-way ANOVA with Tukey’s multiple comparisons test. Sample sizes: Control n=4, HuCNS-SC untreated n=9, HuCNS-SC treated n=9, NB untreated n=15, NB treated n=15.

Animals that were not sacrificed at P180 were maintained until P240. At P240, we were able to assess retinal function with scotopic ERG using a 3.55 log cd * s/m^2 flash. There was no significant difference in a-wave and b-wave amplitudes, however, b-wave amplitudes from the cell-treated groups were higher than the BSS-injected control group. B-wave amplitudes averaged 93.4μV and 83.3μV for NB and HuCNS-SC respectively, BSS-injected controls averaged 59.2uV (**Figure 4**).

Fig 4. No significant functional changes were measured after cell transplantation.

(A) Representative waveforms from BSS-injected, HuCNS-SC®-treated, and NB-treated eyes immediately prior to harvest. (B) Average a-wave and b-wave amplitudes for each

group extracted from waveforms. Sample sizes: n=7 BBS-injected, n=3 HuCNS-SC®-treated, and n=5 NB-treated. There were no statistical differences between the groups. Statistical analysis was performed with a one-way ANOVA with Tukey's multiple comparisons test.

One of the last pieces of data we wanted for the investigational new drug (IND) package was safety. HuCNS-SC are known to have site-appropriate global migration. We wanted to ensure that the cells were not migrating out of the retina. To this end, we harvest whole globes and optic nerves so we could count cells in the optic nerve heads and optic nerves. Sections stained with Ki-67 and STEM121 revealed no cell proliferation in the optic nerve or optic nerve head of any treated or untreated eyes (**Figure 5**). No transplanted cells were found in the optic nerves of any injected eyes. No significant presence of transplanted cells was found in the optic nerve heads of treated eyes with only two cell total being found near the optic nerve head of one NB treated eyes (**Figure 5**, arrows).

Fig 5. No significant cell proliferation or migration was found after cell transplantation.

Representative sections of optic nerve and optic nerve head stained with human cell marker (STEM121, red), cell proliferation marker (Ki-67, green), and nuclear counterstain (DAPI, blue) at P240. Sample sizes: n=8 BBS-injected, n=5 HuCNS-SC®-treated, and n=5 NB-treated. No significant presence of transplanted cells was noted in either the optic nerve or optic nerve head. No cell proliferation was seen in any sample.

DISCUSSION

HuCNS-SC contain self-renewing, multi-potent adult stem cells purified, expanded, and banked from a single human fetal brain tissue procured under GTP through an approved tissue procurement agency from voluntary, consented, unpaid donors.[6] HuCNS-SC are grown as neurospheres with the final product containing cell clusters. While the HuCNS-SC product has shown great retinal neuroprotective capacity in pre-clinical and clinical studies,[7–9,11,14,19,20] we hypothesized that a single-cell formulation would 1) facilitate transplantation with a more consistent cell dose; 2) spread more evenly than cell clusters, covering a larger surface area for improved therapeutic benefit; and 3) be delivered through a smaller subretinal delivery cannula for a safer procedure. Thus, NB, a single-cell formulation of HuCNS-SC, was generated by gentle dissociation of the neurophseres. To grow NB, cells derived from the same master cell bank (MCB) as HuCNS-SC (MCB 5016.M3) were produced in a similar manufacturing process using a closed bioreactor system and formulated as a single-cell suspension, resulting in only two relatively minor changes in the product's chemistry manufacturing and controls (CMC). Since HuCNS-SC had already achieved investigational new drug (IND) approval for a Phase I/II Study entitled Safety and Preliminary Efficacy of Human Central Nervous System Stem Cells (HuCNS-SC) Subretinal Transplantation in Subjects With Geographic Atrophy of Age-Related Macular Degeneration (NCT01632527),[14] we designed a bridging study to demonstrate that NB elicited neuroprotection effects on par or better than the previous cluster formulation, with the goal to get IND approval of NB for macular dystrophies.

Pre-clinical evaluation of HuCNS-SC was performed in a rodent model of retinal degeneration, the RCS rat, as this animal model is FDA approved for retinal cell transplantation for ~~age related macular degeneration~~ (AMD).[18] The RCS rat RPE is dysfunctional due to a mutation in the ~~*Mertk* gene~~; and many studies have shown that transplanting functional RPE in the subretinal space can slow retinal degeneration and restore visual function to this animal model.[21–24] HuCNS-SC showed robust retinal neuroprotection in the RCS rat ~~after~~ surviving at least 8 months ~~after~~ transplantation. Photoreceptors were preserved resulting in improved visual performance and minimal loss of vision long-term.[11] Although the neuroprotection mechanisms remain unclear, additional pre-clinical studies highlight the ability of HuCNS-SC to restore phagocytic function, secrete anti-inflammatory and trophic factors, and promote host RPE cell proliferation.[12,13]

Currently, visual performance measured by optokinetic tracking (OKT) and photoreceptor rescue, measured by outer nuclear layer thickness, are the most clinically relevant outcome measurements in the RCS rat. Our major goal of the bridging study was to compare the performance of HuCNS-SC and NB via these two critical outcomes. At our late time point of P180 (6 months of age, ~22 weeks post-injection), NB-injected eyes had improved visual performance compared to HuCNS-SC-injected eyes signifying improved visual outcomes due to the single-cell formulation. When we looked at the retinal cross-sections to verify cell presence and photoreceptors survival, as expected, at P180, NB-injected eyes had more photoreceptors surviving adjacent to treated areas compared to HuCNS-SC-injected eyes. In trying to determine what lead to the increase in neuroprotection from NB, we assessed cell survival and morphology at P60, only 5

weeks post-injection. We could distinctly see differences in cell morphology in the subretinal space. The HuCNS-SC formulation remained as large clusters, whereas the NB formulation spread out and formed thinner monolayers. We quantified cell spread and demonstrated that the single-cell formulation, NB, increased spread by 2-fold. We suspect that the increase in cell spread resulted in a larger treatment area, thereby increasing visual gains out to P180.

Knowing that unwanted cell migration and proliferation continue to be the largest safety concerns for cell transplantation,[25] we wanted to add safety data to the IND application. To this end, we assessed optic nerve heads and optic nerves for cell migration and proliferation. In these tissues, we did not see any cell proliferation as measured with Ki67 and cells were not observed in the optic nerves. These data are in alignment with other pre-clinical studies that demonstrate only site-specific cell migration and proliferation of HuCNS-SC.[13]

One of the main difficulties we had with these long-term survival studies was the use of cyclosporine. When we started these studies, we provided the animals 210 mg/L ad libidum cyclosporine in the water. This resulted in lethargy, weight loss, ataxia and eventual death of our animals.[26] Once we measured the cyclosporine concentration in the blood, to our surprise, this resulted in levels of 2,100 mg/L, the threshold for the test, when therapeutic doses are 300 mg/L. We transitioned to an escalating dose scheme, beginning with a dose of 52.5 mg/L from P21-P35, followed by a dose of 105 mg/L from P35-P150, and ending with a dose of 157.5mg/L from P150-P240. This allowed us to maintain the therapeutic threshold of 300 mg/L, while keeping our animals healthy and alive out to P240. For future studies involving cyclosporine administration, we

recommend an escalating dose scheme and periodic blood testing to ensure adequate immune suppression and health of the animals.

The goal of this bridging study was to utilize the animal model and outcome measurements that would demonstrate to the FDA that NB performs on par or better than HuCNS-SC. With very few changes to the CMC, and years of pre-clinical and clinical studies demonstrating safety of HuCNS-SC, we have been successful at using these data to achieve IND approval to initiate clinical trials with NB for macular degenerative diseases, including GA-AMD, retinitis pigmentosa, and Stargardts.

REFERENCES

1. Wong, W. L. *et al.* Global prevalence of age-related macular degeneration and disease burden projection for 2020 and 2040: a systematic review and meta-analysis. *Lancet Glob. Health* **2**, e106–e116 (2014).
2. Schultz, N. M., Bhardwaj, S., Barclay, C., Gaspar, L. & Schwartz, J. Global Burden of Dry Age-Related Macular Degeneration: A Targeted Literature Review. *Clin. Ther.* **43**, 1792–1818 (2021).
3. Liao, D. S. *et al.* Complement C3 Inhibitor Pegcetacoplan for Geographic Atrophy Secondary to Age-Related Macular Degeneration: A Randomized Phase 2 Trial. *Ophthalmology* **127**, 186–195 (2020).
4. Csaky, K. G., Miller, J. M. L., Martin, D. F. & Johnson, M. W. Drug Approval for the Treatment of Geographic Atrophy: How We Got Here and Where We Need to Go. *Am. J. Ophthalmol.* **263**, 231–239 (2024).

5. Guimaraes, T. A. C. de, Varela, M. D., Georgiou, M. & Michaelides, M. Treatments for dry age-related macular degeneration: therapeutic avenues, clinical trials and future directions. *Br. J. Ophthalmol.* **106**, 297–304 (2022).
6. Uchida, N. *et al.* Direct isolation of human central nervous system stem cells. *Proc. Natl. Acad. Sci.* **97**, 14720–14725 (2000).
7. Uchida, N. *et al.* Human Neural Stem Cells Induce Functional Myelination in Mice with Severe Dysmyelination. *Sci. Transl. Med.* **4**, 10.1126/scitranslmed.3004371 (2012).
8. Tamaki, S. J. *et al.* Neuroprotection of Host Cells by Human Central Nervous System Stem Cells in a Mouse Model of Infantile Neuronal Ceroid Lipofuscinosis. *Cell Stem Cell* **5**, 310–319 (2009).
9. Piltti, K. M. *et al.* Increasing Human Neural Stem Cell Transplantation Dose Alters Oligodendroglial and Neuronal Differentiation after Spinal Cord Injury. *Stem Cell Rep.* **8**, 1534–1548 (2017).
10. Tsukamoto, A., Uchida, N., Capela, A., Gorba, T. & Huhn, S. Clinical translation of human neural stem cells. *Stem Cell Res. Ther.* **4**, 1–13 (2013).
11. McGill, T. J. *et al.* Transplantation of human central nervous system stem cells – neuroprotection in retinal degeneration. *Eur. J. Neurosci.* **35**, 468–477 (2012).
12. Cuenca, N. *et al.* Phagocytosis of photoreceptor outer segments by transplanted human neural stem cells as a neuroprotective mechanism in retinal degeneration. *Invest. Ophthalmol. Vis. Sci.* **54**, 6745–6756 (2013).

13. McGill, T. J. *et al.* Subretinal Transplantation of Human Central Nervous System Stem Cells Stimulates Controlled Proliferation of Endogenous Retinal Pigment Epithelium. *Transl. Vis. Sci. Technol.* **8**, 43 (2019).
14. Mg, N. *et al.* Effect of Human Central Nervous System Stem Cell Subretinal Transplantation on Progression of Geographic Atrophy Secondary to Nonneovascular Age-Related Macular Degeneration. *Ophthalmol. Retina* **5**, (2021).
15. Surendran, H. *et al.* An improved protocol for generation and characterization of human-induced pluripotent stem cell-derived retinal pigment epithelium cells. *STAR Protoc.* **3**, 101803 (2022).
16. Ryals, R. C. *et al.* A Ketogenic & Low-Protein Diet Slows Retinal Degeneration in rd10 Mice. *Transl. Vis. Sci. Technol.* **9**, 18–18 (2020).
17. Tullis, B. E. *et al.* Sarpogrelate, a 5-HT2A Receptor Antagonist, Protects the Retina From Light-Induced Retinopathy. *Invest. Ophthalmol. Vis. Sci.* **56**, 4560–4569 (2015).
18. Ryals, R. C. *et al.* Long-term Characterization of Retinal Degeneration in Royal College of Surgeons Rats Using Spectral-Domain Optical Coherence Tomography. *Invest. Ophthalmol. Vis. Sci.* **58**, 1378–1386 (2017).
19. Levi, A. D. *et al.* Emerging safety of intramedullary transplantation of human neural stem cells in chronic cervical and thoracic spinal cord injury. *Clin. Neurosurg.* **82**, 562–575 (2018).
20. D, L. *et al.* Clinical Outcomes from a Multi-Center Study of Human Neural Stem Cell Transplantation in Chronic Cervical Spinal Cord Injury. *J. Neurotrauma* (2019) doi:10.1089/neu.2018.5843.

21. Soundararajan, L. *et al.* Allogeneic RPE cell suspension manufactured at scale demonstrating preclinical safety and efficacy led to IND approval. *Npj Regen. Med.* **10**, 1–16 (2025).
22. McGill, T. J. *et al.* Long-Term Efficacy of GMP Grade Xeno-Free hESC-Derived RPE Cells Following Transplantation. *Transl. Vis. Sci. Technol.* **6**, 17–17 (2017).
23. J, D. *et al.* Human RPE Stem Cell-Derived RPE Preserves Photoreceptors in the Royal College of Surgeons Rat: Method for Quantifying the Area of Photoreceptor Sparing. *J. Ocul. Pharmacol. Ther.* (2016) doi:10.1089/jop.2015.0162.
24. Rajendran Nair, D. S. *et al.* Long-Term Transplant Effects of iPSC-RPE Monolayer in Immunodeficient RCS Rats. *Cells* **10**, 2951 (2021).
25. Goldring, C. E. P. *et al.* Assessing the Safety of Stem Cell Therapeutics. *Cell Stem Cell* **8**, 618–628 (2011).
26. Patocka, J., Nepovimova, E., Kuca, K. & Wu, W. Cyclosporine A: Chemistry and Toxicity – A Review. *Curr. Med. Chem.* **28**, 3925–3934 (2021).

SUPPLEMENTAL INFORMATION

Supplemental Fig 1. Morphology and FACS of cells during preparation.

Cell viability was measured to prepare cells for transplantation. Cells were stained with trypan blue and examination under microscope. (A) Representative bright field images of HuCNS-SC® and (B) NB highlighting the different morphology between the cluster and single-cell formulations. (C) Representative FACS profiles illustrating CD133 and Sox2 expression in HuCNS-SCs derived from MCB 5016. Cultured neurospheres were dissociated into single-cell suspensions and stained with antibodies against CD133 and

Sox2. Stained populations are shown as black histograms, with unstained controls displayed in red.

Click here to access/download;Figure;Fig 1 PLOS one.pdf

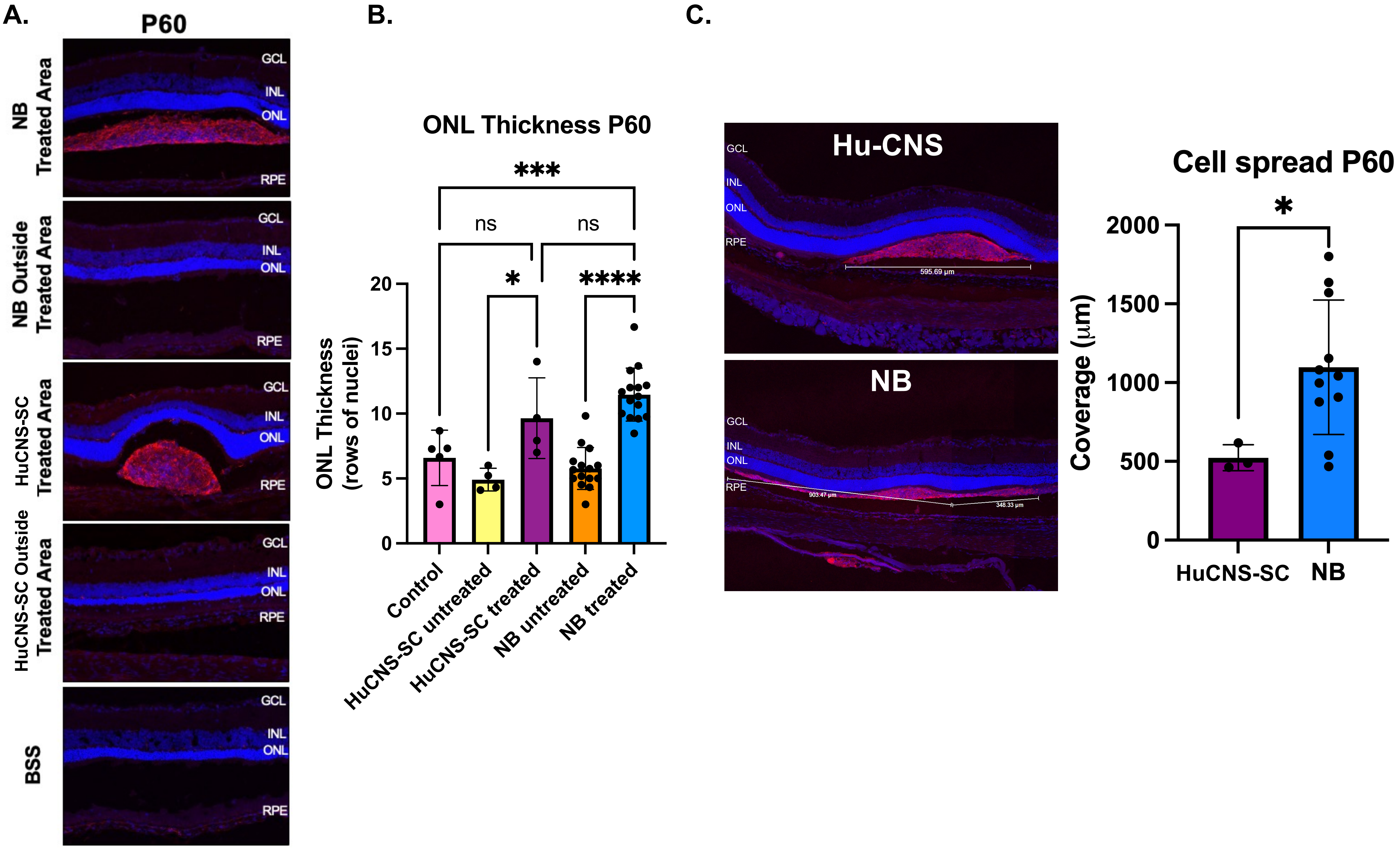

Click here to access/download;Figure;Fig 2 PLOS one.pdf

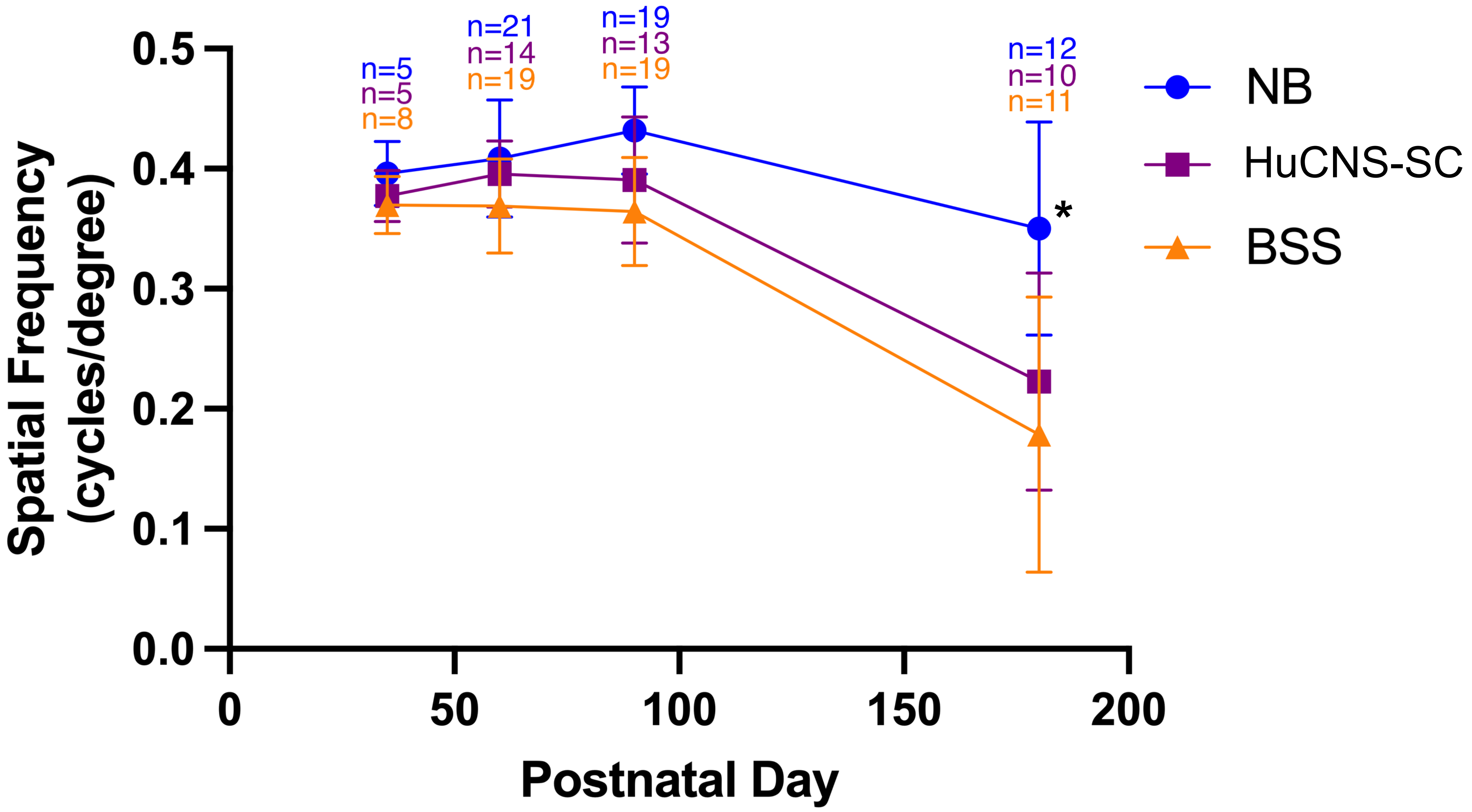

Click here to access/download;Figure;Fig 3 PLOS one.pdf

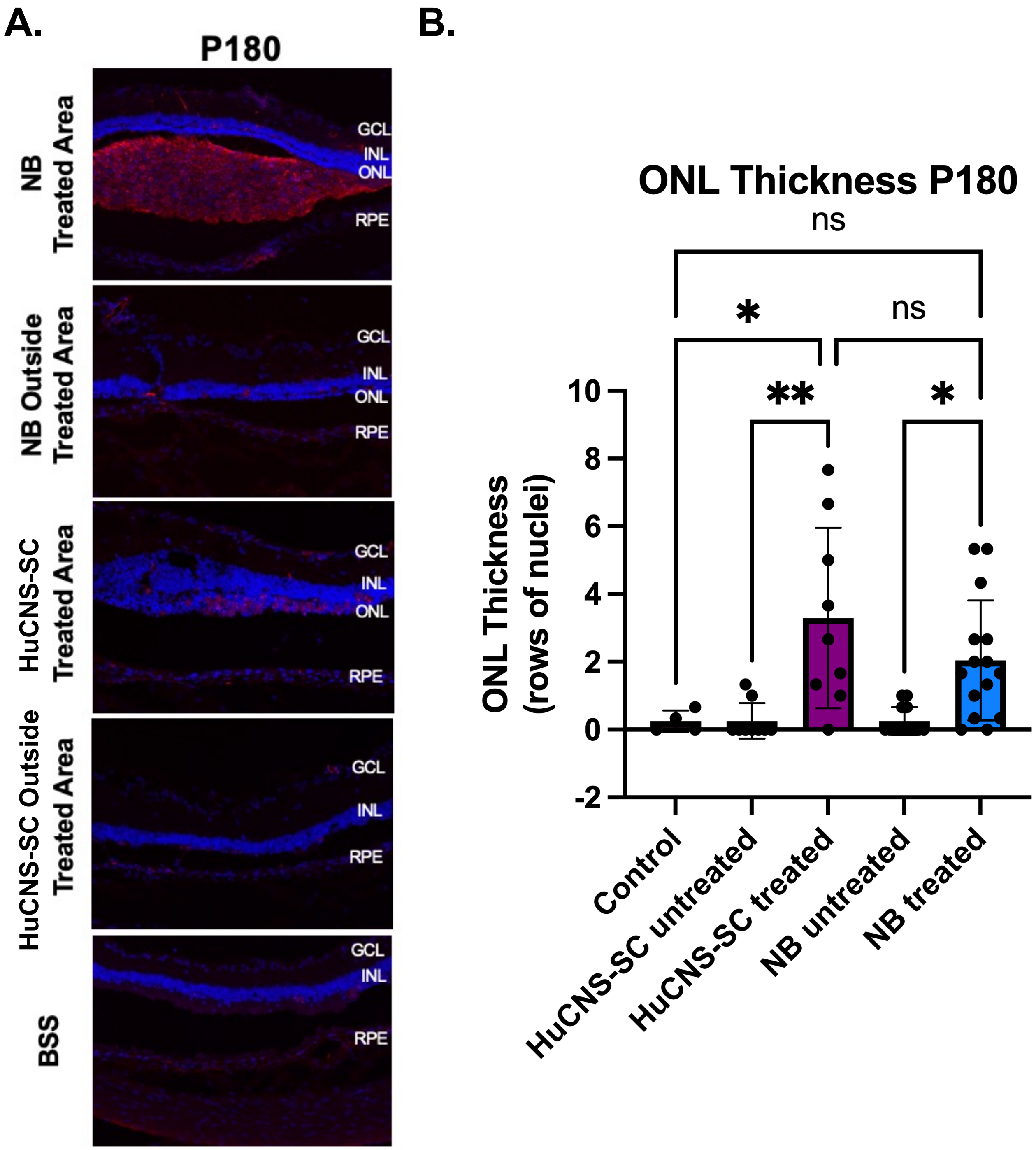

Click here to access/download;Figure;Fig 4 PLOS one.pdf

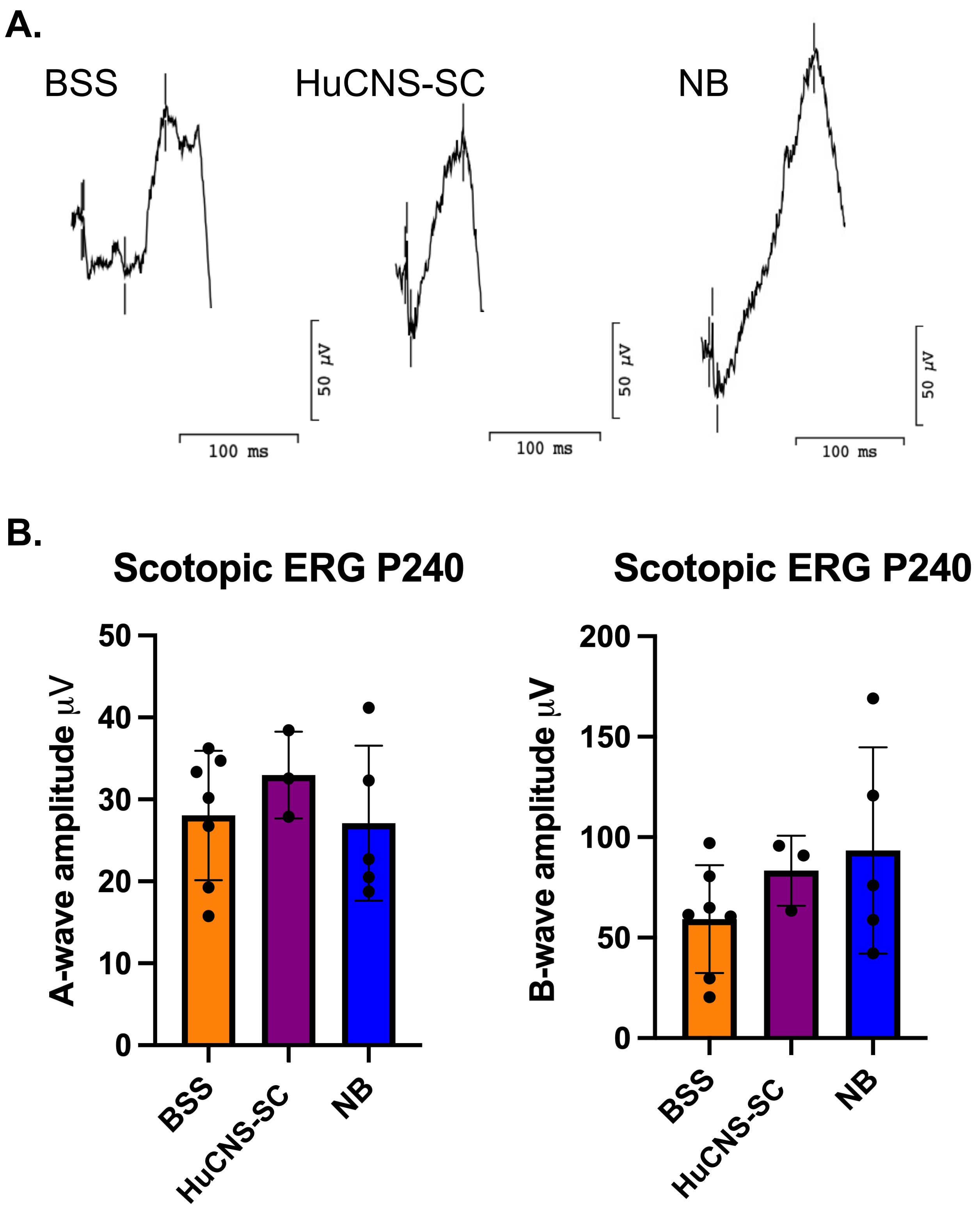

Figure 5

Click here to access/download;Figure;Fig 5 PLOS one.pdf

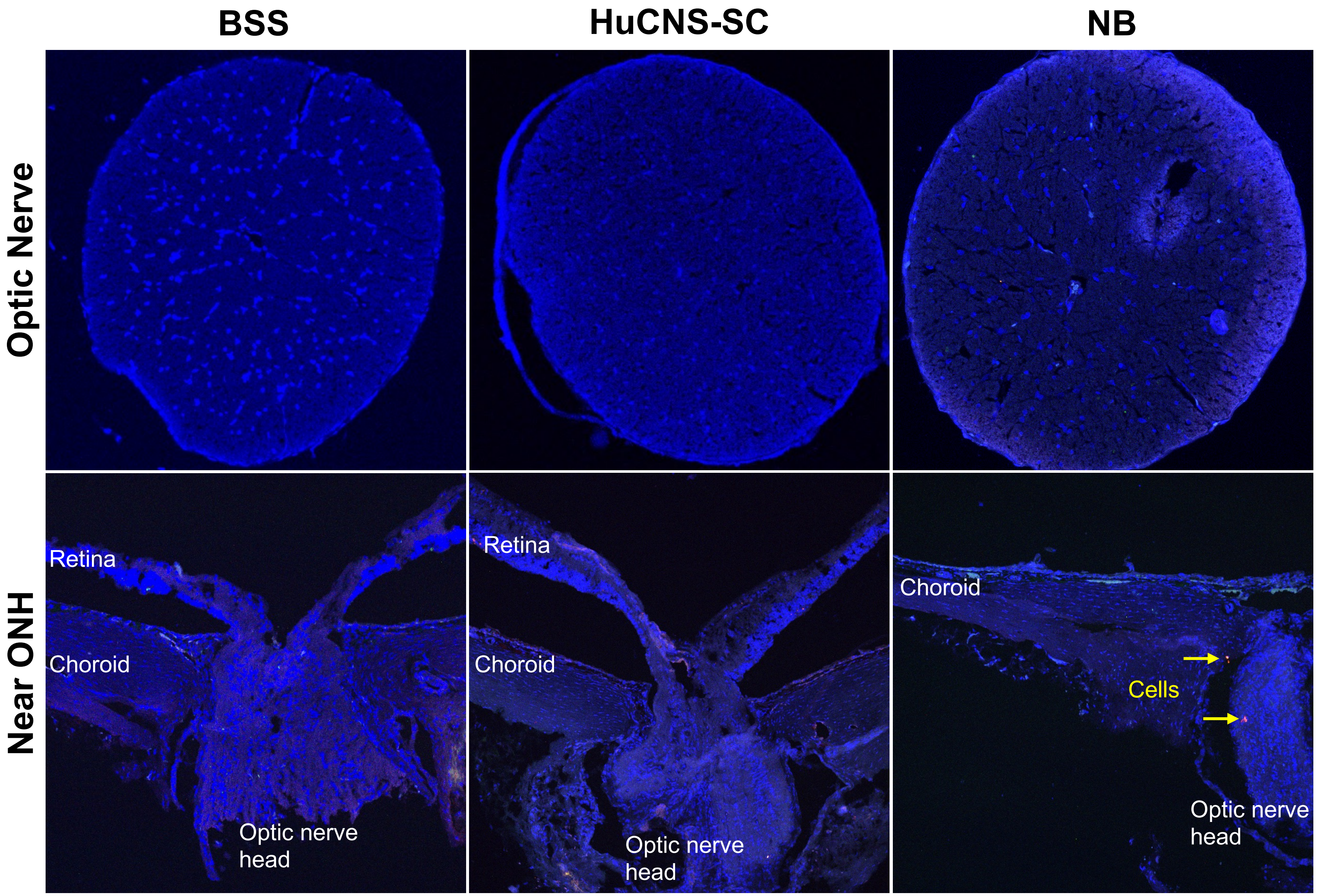

Supporting Figure 1

Click here to access/download
Supporting Information
Supp Fig 1 PLOS one.pdf
