# Supplementary material for: An improved formulation of human neural stem cells for the treatment of geographic atrophy
Source: PLoS One. 2026 Aug 10;21(8):e0355552. doi: 10.1371/journal.pone.0355552 (PMC13456245; doi:10.1371/journal.pone.0355552)
Supplement: Peer review history

# Peer review: Decision Letter 0

**DOI:** 10.1371/journal.pone.0355552.r001

**Authors:** Mohd Akbar Bhat

10 May 2026

PONE-D-25-67955

An improved formulation of human neural stem cells for the treatment of geographic atrophy

PLOS One

Dear Dr. Ryals,

Thank you for submitting your manuscript to PLOS ONE. After careful consideration, we feel that it has merit but does not fully meet PLOS ONE’s publication criteria as it currently stands. Therefore, we invite you to submit a revised version of the manuscript that addresses the points raised during the review process.

==============================

**The authors are requested to read the reviewers comments carefully and incorporate all the recommended changes.**

==============================

Please submit your revised manuscript by Jun 24 2026 11:59PM. If you will need more time than this to complete your revisions, please reply to this message or contact the journal office at plosone@plos.org. When you're ready to submit your revision, log on to https://www.editorialmanager.com/pone/ and select the 'Submissions Needing Revision' folder to locate your manuscript file.

Please include the following items when submitting your revised manuscript:

- A letter that responds to each point raised by the academic editor and reviewer(s). You should upload this letter as a separate file labeled 'Response to Reviewers'.
- A marked-up copy of your manuscript that highlights changes made to the original version. You should upload this as a separate file labeled 'Revised Manuscript with Track Changes'.
- An unmarked version of your revised paper without tracked changes. You should upload this as a separate file labeled 'Manuscript'.

If you would like to make changes to your financial disclosure, please include your updated statement in your cover letter. Guidelines for resubmitting your figure files are available below the reviewer comments at the end of this letter.

If applicable, we recommend that you deposit your laboratory protocols in protocols.io to enhance the reproducibility of your results. Protocols.io assigns your protocol its own identifier (DOI) so that it can be cited independently in the future. For instructions see: https://journals.plos.org/plosone/s/submission-guidelines#loc-laboratory-protocols. Additionally, PLOS ONE offers an option for publishing peer-reviewed Lab Protocol articles, which describe protocols hosted on protocols.io. Read more information on sharing protocols at https://plos.org/protocols?utm_medium=editorial-email&utm_source=authorletters&utm_campaign=protocols.

As the corresponding author, your ORCID iD is verified in the submission system and will appear in the published article. PLOS supports the use of ORCID, and we encourage all coauthors to register for an ORCID iD and use it as well. Please encourage your coauthors to verify their ORCID iD within the submission system before final acceptance, as unverified ORCID iDs will not appear in the published article. *Only*  the individual author can complete the verification step; PLOS staff *cannot*  verify ORCID iDs on behalf of authors.

We look forward to receiving your revised manuscript.

Kind regards,

Mohd Akbar Bhat, Ph.D.

Academic Editor

PLOS One

Journal Requirements:

When submitting your revision, we need you to address these additional requirements.

1. Please ensure that your manuscript meets PLOS ONE's style requirements, including those for file naming. The PLOS ONE style templates can be found at https://journals.plos.org/plosone/s/file?id=wjVg/PLOSOne_formatting_sample_main_body.pdf and https://journals.plos.org/plosone/s/file?id=ba62/PLOSOne_formatting_sample_title_authors_affiliations.pdf

2. To comply with PLOS One submissions requirements, in your Methods section, please provide additional information regarding the experiments involving animals and ensure you have included details on (1) methods of sacrifice, (2) methods of anesthesia and/or analgesia, and (3) efforts to alleviate suffering.

3. Please note that PLOS One has specific guidelines on code sharing for submissions in which author-generated code underpins the findings in the manuscript. In these cases, we expect all author-generated code to be made available without restrictions upon publication of the work. Please review our guidelines at https://journals.plos.org/plosone/s/materials-and-software-sharing#loc-sharing-code and ensure that your code is shared in a way that follows best practice and facilitates reproducibility and reuse.

4. We note that the grant information you provided in the ‘Funding Information’ and ‘Financial Disclosure’ sections do not match.

When you resubmit, please ensure that you provide the correct grant numbers for the awards you received for your study in the ‘Funding Information’ section.

5. Thank you for stating in your Funding Statement:

[This work was supported by CIRM TRAN1-11300 (TL, RCR), NIH grant P30-EY026877 (TL), NIH P30 EY010572 core grant, the Malcolm M. Marquis, MD Endowed Fund for Innovation, and an unrestricted grant from Research to Prevent Blindness (TL, RCR). BOCO Silicon Valley provided the hNSCs.].

Please provide an amended statement that declares *all* the funding or sources of support (whether external or internal to your organization) received during this study, as detailed online in our guide for authors at http://journals.plos.org/plosone/s/submit-now.  Please also include the statement “There was no additional external funding received for this study.” in your updated Funding Statement.

Please include your amended Funding Statement within your cover letter. We will change the online submission form on your behalf.

6. Please expand the acronyms “CIRM” and “NIH” (as indicated in your financial disclosure) so that it states the name of your funders in full.

This information should be included in your cover letter; we will change the online submission form on your behalf.

7. Please remove any funding-related text from the manuscript. Funding information should not appear in any section or other areas of your manuscript. We will only publish funding information present in the Funding Statement section of the online submission form.

8. Thank you for stating the following in the Financial Disclosure section:

[This work was supported by CIRM TRAN1-11300 (TL, RCR), NIH grant P30-EY026877 (TL), NIH P30 EY010572 core grant, the Malcolm M. Marquis, MD Endowed Fund for Innovation, and an unrestricted grant from Research to Prevent Blindness (TL, RCR). BOCO Silicon Valley provided the hNSCs.].

We note that you may have received funding from a commercial source: BOCO Silicon Valley

Please provide an amended Competing Interests Statement that explicitly states this commercial funder, along with any other relevant declarations relating to employment, consultancy, patents, products in development, marketed products, etc.

Within this Competing Interests Statement, please confirm that this does not alter your adherence to all PLOS ONE policies on sharing data and materials by including the following statement: "This does not alter our adherence to PLOS ONE policies on sharing data and materials.” (as detailed online in our guide for authors http://journals.plos.org/plosone/s/competing-interests).  If there are restrictions on sharing of data and/or materials, please state these. Please note that we cannot proceed with consideration of your article until this information has been declared.

Please include your amended Competing Interests Statement within your cover letter. We will change the online submission form on your behalf.

If the reviewer comments include a recommendation to cite specific previously published works, please review and evaluate these publications to determine whether they are relevant and should be cited. There is no requirement to cite these works unless the editor has indicated otherwise.

[Note: HTML markup is below. Please do not edit.]

Reviewers' comments:

Reviewer's Responses to Questions

**Comments to the Author**

1. Is the manuscript technically sound, and do the data support the conclusions?

The manuscript must describe a technically sound piece of scientific research with data that supports the conclusions. Experiments must have been conducted rigorously, with appropriate controls, replication, and sample sizes. The conclusions must be drawn appropriately based on the data presented.

Reviewer #1: Yes

Reviewer #2: Partly

**********

2. Has the statistical analysis been performed appropriately and rigorously?

Reviewer #1: Yes

Reviewer #2: Yes

**********

3. Have the authors made all data underlying the findings in their manuscript fully available?

The PLOS Data policy requires authors to make all data underlying the findings described in their manuscript fully available without restriction, with rare exception (please refer to the Data Availability Statement in the manuscript PDF file). The data should be provided as part of the manuscript or its supporting information, or deposited to a public repository. For example, in addition to summary statistics, the data points behind means, medians and variance measures should be available. If there are restrictions on publicly sharing data—e.g. participant privacy or use of data from a third party—those must be specified.

Reviewer #1: Yes

Reviewer #2: Yes

**********

4. Is the manuscript presented in an intelligible fashion and written in standard English?

PLOS ONE does not copyedit accepted manuscripts, so the language in submitted articles must be clear, correct, and unambiguous. Any typographical or grammatical errors should be corrected at revision, so please note any specific errors here.

Reviewer #1: Yes

Reviewer #2: No

**********

5. Review Comments to the Author

Please use the space provided to explain your answers to the questions above. You may also include additional comments for the author, including concerns about dual publication, research ethics, or publication ethics. (Please upload your review as an attachment if it exceeds 20,000 characters)

Reviewer #1: Dear authors

I had the privilege of reviewing your manuscript.

A few suggestions below:

Abstract:

Results: …ONL thickness was Increased in areas adjacent to the injected cells…

Maybe a better way to describe it would be to say that the ONL thickness loss was LOWER than the untreated areas, since in RCS rats the degeneration is progressive and the thinning of the layers happens inexorably. THe stem cells may have SLOWERED the ONL cell loss compared to untreated areas, and that’s consistent with the literature.

Delaying the progression of the degeneration should be the message.

Methods

Safety

Since this preclinical trial aims at acquiring FDA approval for a clinical trial, the FDA formerly required samples for all organs and tissues to demonstrate lack of cell migration and tumor formation outside the eye and visual axis.

For your study, the FDA required only samples for the eye and optic nerve?

Early assessment

During subretinal injection its very frequent to find iatrogenic damage to the eye tissues related to the injection procedure.

It is routine to discard animals due to complications.

Did the group discarded rats because of inadvertent bleeding, retinal detachment or infection post injection?

I could not find this data on the paper, would be good to state which complications the rate of this the group found during the study.

After injection the volume and thickness of the injected cells in the subretinal space tends to diminish over time, this was viewed in this study?

No live fundus photos, autofluorescence, fluorescein angiography and OCT direct images of the cells in the subretinal space were acquired in the study.

Why the author opted only for the post Morten analysis?

Best

Reviewer #2: The authors present an interesting study on the therapeutic potential of human neural stem cells for retinal degenerative diseases. The topic is timely and highly relevant to current clinical practice and ongoing research, and the manuscript offers several valuable insights.

However, the paper would benefit from revisions, particularly in its overall structure, figure clarity, and writing style. In addition, some references appear to be missing and should be addressed. Improving these aspects will enhance clarity, readability, and alignment with the standards expected for scientific publication.

I have included detailed comments and suggestions in the attached revision file, which I hope will assist in strengthening the manuscript. Thank you for your contribution to the field, and I look forward to reviewing a revised version.

**********

6. PLOS authors have the option to publish the peer review history of their article (what does this mean?). If published, this will include your full peer review and any attached files.

If you choose “no”, your identity will remain anonymous but your review may still be made public.

**Do you want your identity to be public for this peer review?**  For information about this choice, including consent withdrawal, please see our Privacy Policy.

Reviewer #1: **Yes:**  Rodrigo Antonio Brant Fernandes

Reviewer #2: No

**********

[NOTE: If reviewer comments were submitted as an attachment file, they will be attached to this email and accessible via the submission site. Please log into your account, locate the manuscript record, and check for the action link "View Attachments". If this link does not appear, there are no attachment files.]

To ensure your figures meet our technical requirements, please review our figure guidelines: https://journals.plos.org/plosone/s/figures

You may also use PLOS’s free figure tool, NAAS, to help you prepare publication quality figures: https://journals.plos.org/plosone/s/figures#loc-tools-for-figure-preparation.

NAAS will assess whether your figures meet our technical requirements by comparing each figure against our figure specifications.

Attachment Submitted filename: PONE-D-25-67955 (for revision).pdf

# Author comment: Author response to Decision Letter 1

**DOI:** 10.1371/journal.pone.0355552.r002

23 Jun 2026

Response to Reviewers

PONE-D-25-67955

We greatly appreciate the editor and the reviewers for their constructive feedback. It has improved the quality of the manuscript. We appreciate the time and effort in considering this manuscript for publication.

Editor:

#1. Please ensure that your manuscript meets PLOS ONE's style requirements, including those for file naming. The PLOS ONE style templates can be found at https://journals.plos.org/plosone/s/file?id=wjVg/PLOSOne_formatting_sample_main_body.pdf and https://journals.plos.org/plosone/s/file?id=ba62/PLOSOne_formatting_sample_title_authors_affiliations.pdf

Headers, sub-headers, figure references, in text citations, and references have been changed to conform to the PLOS ONE standards.

#2. To comply with PLOS One submissions requirements, in your Methods section, please provide additional information regarding the experiments involving animals and ensure you have included details on (1) methods of sacrifice, (2) methods of anesthesia and/or analgesia, and (3) efforts to alleviate suffering.

Details of anesthesia, post-operative care, and methods of sacrifice have been added to the Animals subsection of Methods.

#3. Please note that PLOS One has specific guidelines on code sharing for submissions in which author-generated code underpins the findings in the manuscript. In these cases, we expect all author-generated code to be made available without restrictions upon publication of the work. Please review our guidelines at https://journals.plos.org/plosone/s/materials-and-software-sharing#loc-sharing-code and ensure that your code is shared in a way that follows best practice and facilitates reproducibility and reuse.

No author generated code was used for this research or manuscript.

#4. We note that the grant information you provided in the ‘Funding Information’ and ‘Financial Disclosure’ sections do not match.

When you resubmit, please ensure that you provide the correct grant numbers for the awards you received for your study in the ‘Funding Information’ section.

Please see answer to comment #5.

#5. Thank you for stating in your Funding Statement:

[This work was supported by CIRM TRAN1-11300 (TL, RCR), NIH grant P30-EY026877 (TL), NIH P30 EY010572 core grant, the Malcolm M. Marquis, MD Endowed Fund for Innovation, and an unrestricted grant from Research to Prevent Blindness (TL, RCR). BOCO Silicon Valley provided the hNSCs.].

Please provide an amended statement that declares *all* the funding or sources of support (whether external or internal to your organization) received during this study, as detailed online in our guide for authors at http://journals.plos.org/plosone/s/submit-now. Please also include the statement “There was no additional external funding received for this study.” in your updated Funding Statement.

Please include your amended Funding Statement within your cover letter. We will change the online submission form on your behalf.

The funding statement has been added to the cover letter. Please use this funding statement in any appropriate places in the manuscript.

#6. Please expand the acronyms “CIRM” and “NIH” (as indicated in your financial disclosure) so that it states the name of your funders in full.

CIRM and NIH have been spelled out in the funding statement within the cover letter.

#7. Please remove any funding-related text from the manuscript. Funding information should not appear in any section or other areas of your manuscript. We will only publish funding information present in the Funding Statement section of the online submission form.

The funding information has been removed from the manuscript file.

#8. Thank you for stating the following in the Financial Disclosure section:

[This work was supported by CIRM TRAN1-11300 (TL, RCR), NIH grant P30-EY026877 (TL), NIH P30 EY010572 core grant, the Malcolm M. Marquis, MD Endowed Fund for Innovation, and an unrestricted grant from Research to Prevent Blindness (TL, RCR). BOCO Silicon Valley provided the hNSCs.].

We note that you may have received funding from a commercial source: BOCO Silicon Valley

Please provide an amended Competing Interests Statement that explicitly states this commercial funder, along with any other relevant declarations relating to employment, consultancy, patents, products in development, marketed products, etc.

Within this Competing Interests Statement, please confirm that this does not alter your adherence to all PLOS ONE policies on sharing data and materials by including the following statement: "This does not alter our adherence to PLOS ONE policies on sharing data and materials.” (as detailed online in our guide for authors http://journals.plos.org/plosone/s/competing-interests). If there are restrictions on sharing of data and/or materials, please state these. Please note that we cannot proceed with consideration of your article until this information has been declared.

Please include your amended Competing Interests Statement within your cover letter. We will change the online submission form on your behalf.

If the reviewer comments include a recommendation to cite specific previously published works, please review and evaluate these publications to determine whether they are relevant and should be cited. There is no requirement to cite these works unless the editor has indicated otherwise.

The amended Competing Interests Statement is within the cover letter. There was no request to cite specific published works.

Reviewer #1:

#1. Results: …ONL thickness was Increased in areas adjacent to the injected cells…

Maybe a better way to describe it would be to say that the ONL thickness loss was LOWER than the untreated areas, since in RCS rats the degeneration is progressive and the thinning of the layers happens inexorably. THe stem cells may have SLOWERED the ONL cell loss compared to untreated areas, and that’s consistent with the literature.

Delaying the progression of the degeneration should be the message.

We have altered our phrasing to clarify that the treatment resulted in delayed disease progression and not an increase in the number of photoreceptors.

#2. Since this preclinical trial aims at acquiring FDA approval for a clinical trial, the FDA formerly required samples for all organs and tissues to demonstrate lack of cell migration and tumor formation outside the eye and visual axis.

For your study, the FDA required only samples for the eye and optic nerve?

Examinations of all organs and tissues for lack of cell migration and tumor formation were performed extensively in other studies. The FDA was satisfied with those extensive safety studies with HuCNS-SC and the addition of these safety data in the bridging study.

#3. During subretinal injection its very frequent to find iatrogenic damage to the eye tissues related to the injection procedure. It is routine to discard animals due to complications.

Did the group discarded rats because of inadvertent bleeding, retinal detachment or infection post injection? I could not find this data on the paper, would be good to state which complications the rate of this the group found during the study.

Injections did not result in infections, bleeding, or retinal detachment post-injection. Eyes were excluded if the needle punctured through the retina allowing cells to go into the vitreous cavity. These were documented as “blow-throughs” and removed from the study. This text was added to the Injections subsection of the Methods.

#4. After injection the volume and thickness of the injected cells in the subretinal space tends to diminish over time, this was viewed in this study?

Yes, this can be observed if you compare early timepoint immunofluorescence (IF) images (Fig 1) to later timepoint IF images (Fig 3). The volume and thickness of injected cells diminished significantly over time. We did not utilize OCT to image the cells over time. The injections are placed far in the periphery and often OCT cannot properly capture the transplanted cells.

#5. No live fundus photos, autofluorescence, fluorescein angiography and OCT direct images of the cells in the subretinal space were acquired in the study.

Why the author opted only for the post Morten analysis?

Please see answer to question #4. Additionally, this study was focused on FDA requirements. Currently, visual performance measured by optokinetic tracking (OKT) and photoreceptor rescue, measured by outer nuclear layer thickness, are the most clinically relevant outcome measurements in the RCS rat. Our main goal of the bridging study was to compare the performance of HuCNS-SC and NB via these two critical outcomes.

Reviewer #2:

#1. Line 62: Couldn’t understand this sentence

The phrasing was changed to increase clarity. Text is now “Currently, there are two FDA approved complement inhibitor therapies, pegcetacoplan and avacincaptad pegol, for GA-AMD.”

#2. Line 65: Rewrite

This sentence has been rewritten. Text is now “One example, human central nervous system stem cells (HuCNS-SC), grown as neurospheres, have been in clinical development as a neuroprotective agent to slow the progression of GA-AMD for decades [6].”

#3. Line 69-75: Reference missing

The reference has been added. It is reference #6.

#4 Line 77-80: Reference missing

The reference has been added to the manuscript and reference list. It is reference #11.

#5. Line 89: Rephrase to “helped minimize long-term vision loss”

This sentence has been rephrased as suggested.

#6. Line 95: Use abbreviation “GA-AMD”

The full text has been changed to the abbreviation.

#7. Line 113: What was the rationale for using both in the study

It’s common practice to perform a bridging study comparing cells made with previous manufacturing protocols to cells made using the new manufacturing protocols. The FDA seems satisfied with this comparison to validate the new product. Text now reads “This bridging study comparing the neuroprotective effects of the old product, HuCNS-SC, and the new product, NB, will be used in an investigational new drug (IND) enabling study to evaluate NB in clinical trials for macular retinal degeneration.”

#8. Line 113: Full form here before abbreviation

“Investigational new drug” was added before IND.

#9. Line 118: Just RCS

The full was form deleted.

#10. Line 118: Place also to mention

Location of the RRRC, University of Missouri Columbia, MO was added.

#11. Line 131: typo neurospheres

The typo was corrected.

#12. Line 180: Add company

The Company name, Takara Bio, was added.

#13. Line 183: typo ImageJ

The typo was corrected.

#14. Line 183: delete “the”

The extra “the” was deleted.

#15. Line 219: Indicate the red and brown pattern on the graph in Fig S1C

“Unstained control”, “CD133+” and “SOX2+” labels were added to differentiate the red and brown histograms. Font colors were also updated.

#16. Line 225-230: Authors here are putting the reference but they should generally reference the results

Reference is used to describe the known pattern of retinal degeneration in untreated RCS rats, as opposed to our controls which were vehicle injected. The word “typically” was added in the text to help with the distinction.

#17. Line 234: Terms outside treated area and untreated both used, need to clarify on Fig 1A

The labels were changed to clarify outside treated area. All necessary labels read “outside treated area.”

#18. Line 238: In Fig1B there is some difference between HuCNS-SC untreated and NB untreated, is this due to animal numbers

We performed the proper statistical analysis comparing these two groups and there was no significant difference. Without any statistical difference, the difference you are referring to is within normal variation. There is variation in retinal degeneration patterns so animal numbers can contribute to differences.

#19. Line 247: For clarity of Fig 1C are there magnified images of both?

The purpose of the images in Fig 1C is to show the full extent of the bleb. These images were taken at a lower magnification in order to include the full bleb.

#20. Line 291: Fig 3B needs clarity and also there is no labeling inside figures. HuCNS-SC shows more ONL thickness than NB.

The labels were changed for clarity as in Fig 1B. The average ONL thickness was slightly higher for HuCNS-SC than for NB, however this difference was not statistically significant.

#21. Line 307: No significance bars in graph. A-wave slightly higher in HuCNS-SC treated compared to NB, authors only mention b-wave

Significance bars have been added to the graphs. No significance was found between the groups for either a- or b-wave. We have added a-wave values to the text. The text now reads, “There was no significant difference in a-wave and b-wave amplitudes. A-wave amplitudes averaged 27.1µV and 33.0µV for NB and HuCNS-SC respectively, whereas BSS-injected controls averaged 28.0µV (Fig 4). Notably, b-wave amplitudes from the cell-treated groups were higher than the BSS-injected control group. B-wave amplitudes averaged 93.4µV and 83.3µV for NB and HuCNS-SC respectively, whereas BSS-injected controls averaged 59.2µV (Fig 4).”

#22. Line 312: Needs to be checked and redefined

No statistically significant differences were found between the groups so we retained the title of the figure.

#23. Line 320: highlighted text

For clarification, text now reads “HuCNS-SC are known to have site-appropriate global migration within the brain. When injected into the hippocampus, HuCNS-SCs migrated up to ±1.7 mm from the injection site [11].”

#24. Line 324: Fig 5 needs magnified image of Ki-67 and STEM121 colabeled

Magnified images have been added as an inset, no co-labeling of red and green was found. Only STEM121+ cells are present.

#25. Line 333: highlighted text

Added company information for the antibodies.

#26. Line 340: Full form not abbreviation

The full form, Good Tissue Practices, was added before the abbreviation.

#27. Line 349: typo neurospheres

The typo was corrected.

#28. Line 356: crossed out (HuCNS-SC)

This text was deleted.

#29. Line 362: crossed out “age related macular degeneration”

This text was deleted.

#30. Line 363: role of gene to mention and can describe this in introduction

The effect of the gene mutation was specified for detail and clarity. The text now reads “The RCS rat RPE is dysfunctional due to a mutation in the Mertk gene, which renders the RPE unable to properly phagocytose outer segments.”

#31. Line 366: strike through “after”

The word “after” was deleted.

#32. Line 366: replace “after” with “post-“

The phrase was changed to “post-transplantation”.

#33. Line 368: rephrase to “loss of vision in the long-term”

The sentence was rephrased as suggested.

#34. Line 376-378: Needs rewrite as claiming NB superior to HuCNS-SC is contradictory to results

The text now reads “When we looked at the retinal cross-sections to verify cell presence and photoreceptors survival, NB-injected eyes and HuCNS-SC-injected eyes had a similar number of surviving photoreceptors adjacent to treated areas demonstrating comparable effectiveness (Fig 3).”

#35. Line 380-381: Check with reference to Fig 3A and B shown

The figure reference was corrected.

#36. Line 392-393: Correct this as STEM121 mentioned previously for checking cell proliferation (Fig 5)

STEM 121 is a human cell marker and was used to check for presence of cells not for proliferation. We used Ki67 as our cell proliferation marker. Text now reads “In these tissues, we did not see any cell proliferation (Ki67+ labeling) and cells (STEM121+ labeling) were not observed in the optic nerves.”

#37. Line 397: highlighted text

210 mg/L refers to the concentration of cyclosporine in the water supplied ad-libidum to the experimental animals. We checked all units referenced and made sure they were correct.

#38. Line 404: Is the therapeutic threshold determined in the study or by reference. Study shows usage of 210mg/mL that is below threshold.

210 mg/L refers to the concentration of cyclosporine in the water given to experimental animals. The therapeutic dose of 300 ng/mL was mislabeled as mg/L and refers to blood concentration (not a given dose). Phrasing and labels have been changed to clarify between administered doses, measured as

Attachment Submitted filename: Response to Reviewers.docx

# Peer review: Decision Letter 1

**DOI:** 10.1371/journal.pone.0355552.r003

**Authors:** Mohd Akbar Bhat

23 Jul 2026

An improved formulation of human neural stem cells

for the treatment of geographic atrophy

PONE-D-25-67955R1

Dear Dr. Ryals

We’re pleased to inform you that your manuscript has been judged scientifically suitable for publication and will be formally accepted for publication once it meets all outstanding technical requirements.

Within one week, you’ll receive an e-mail detailing the required amendments. When these have been addressed, you’ll receive a formal acceptance letter and your manuscript will be scheduled for publication.

An invoice will be generated when your article is formally accepted. Please note, if your institution has a publishing partnership with PLOS and your article meets the relevant criteria, all or part of your publication costs will be covered. Please make sure your user information is up-to-date by logging into Editorial Manager at Editorial Manager® and clicking the ‘Update My Information' link at the top of the page. For questions related to billing, please contact billing support.

If your institution or institutions have a press office, please notify them about your upcoming paper to help maximize its impact. If they’ll be preparing press materials, please inform our press team as soon as possible -- no later than 48 hours after receiving the formal acceptance. Your manuscript will remain under strict press embargo until 2 pm Eastern Time on the date of publication. For more information, please contact onepress@plos.org.

Kind regards,

Mohd Akbar Bhat, Ph.D.

Academic Editor

PLOS One

Additional Editor Comments (optional):

Reviewers' comments:

Reviewer's Responses to Questions

**Comments to the Author**

1. If the authors have adequately addressed your comments raised in a previous round of review and you feel that this manuscript is now acceptable for publication, you may indicate that here to bypass the “Comments to the Author” section, enter your conflict of interest statement in the “Confidential to Editor” section, and submit your "Accept" recommendation.

Reviewer #1: All comments have been addressed

Reviewer #2: All comments have been addressed

**********

2. Is the manuscript technically sound, and do the data support the conclusions?

The manuscript must describe a technically sound piece of scientific research with data that supports the conclusions. Experiments must have been conducted rigorously, with appropriate controls, replication, and sample sizes. The conclusions must be drawn appropriately based on the data presented.

Reviewer #1: Yes

Reviewer #2: Yes

**********

3. Has the statistical analysis been performed appropriately and rigorously?

Reviewer #1: Yes

Reviewer #2: Yes

**********

4. Have the authors made all data underlying the findings in their manuscript fully available?

The PLOS Data policy requires authors to make all data underlying the findings described in their manuscript fully available without restriction, with rare exception (please refer to the Data Availability Statement in the manuscript PDF file). The data should be provided as part of the manuscript or its supporting information, or deposited to a public repository. For example, in addition to summary statistics, the data points behind means, medians and variance measures should be available. If there are restrictions on publicly sharing data—e.g. participant privacy or use of data from a third party—those must be specified.

Reviewer #1: Yes

Reviewer #2: Yes

**********

5. Is the manuscript presented in an intelligible fashion and written in standard English?

PLOS ONE does not copyedit accepted manuscripts, so the language in submitted articles must be clear, correct, and unambiguous. Any typographical or grammatical errors should be corrected at revision, so please note any specific errors here.

Reviewer #1: Yes

Reviewer #2: Yes

**********

6. Review Comments to the Author

Please use the space provided to explain your answers to the questions above. You may also include additional comments for the author, including concerns about dual publication, research ethics, or publication ethics. (Please upload your review as an attachment if it exceeds 20,000 characters)

Reviewer #1: Dear authors

I had the privilege to review the manuscript after the suggestions.

All my comments were addressed.

Reviewer #2: The authors have addressed all of the reviewers' comments satisfactorily, and I am pleased with the revisions made. The manuscript has been significantly improved and is now suitable for publication in its current form.

**********

7. PLOS authors have the option to publish the peer review history of their article (what does this mean?). If published, this will include your full peer review and any attached files.

If you choose “no”, your identity will remain anonymous but your review may still be made public.

**Do you want your identity to be public for this peer review?**  For information about this choice, including consent withdrawal, please see our Privacy Policy.

Reviewer #1: **Yes:**  rodrigo Antonio brant Fernandez

Reviewer #2: No

**********

# Editor report: Acceptance letter

**DOI:** 10.1371/journal.pone.0355552.r004

**Authors:** Mohd Akbar Bhat

PONE-D-25-67955R1

PLOS One

Dear Dr. Ryals,

I'm pleased to inform you that your manuscript has been deemed suitable for publication in PLOS One. Congratulations! Your manuscript is now being handed over to our production team.

At this stage, our production department will prepare your paper for publication. This includes ensuring the following:

* All references, tables, and figures are properly cited

* All relevant supporting information is included in the manuscript submission,

* There are no issues that prevent the paper from being properly typeset

You will receive further instructions from the production team, including instructions on how to review your proof when it is ready. Please keep in mind that we are working through a large volume of accepted articles, so please give us a few days to review your paper and let you know the next and final steps.

Lastly, if your institution or institutions have a press office, please let them know about your upcoming paper now to help maximize its impact. If they'll be preparing press materials, please inform our press team within the next 48 hours. Your manuscript will remain under strict press embargo until 2 pm Eastern Time on the date of publication. For more information, please contact onepress@plos.org.

You will receive an invoice from PLOS for your publication fee after your manuscript has reached the completed accept phase. If you receive an email requesting payment before acceptance or for any other service, this may be a phishing scheme. Learn how to identify phishing emails and protect your accounts at https://explore.plos.org/phishing.

If we can help with anything else, please email us at customercare@plos.org.

Thank you for submitting your work to PLOS ONE and supporting open access.

Kind regards,

PLOS ONE Editorial Office Staff

on behalf of

Dr. Mohd Akbar Bhat

Academic Editor

PLOS One
